# The Understanding of the Metazoan Skeletal System, Based on the Initial Discoveries with Siliceous and Calcareous Sponges

**DOI:** 10.3390/md15060172

**Published:** 2017-06-12

**Authors:** Werner E. G. Müller, Heinz C. Schröder, Xiaohong Wang

**Affiliations:** ERC Advanced Investigator Grant Research Group at the Institute for Physiological Chemistry, University Medical Center of the Johannes Gutenberg University, Duesbergweg 6, D-55128 Mainz, Germany; hschroed@uni-mainz.de

**Keywords:** enzyme-mediated biomineral formation, bone-hydroxyapatite, biosilica, calcium carbonate bio-seeds, alkaline phosphatase, carbonic anhydrase, inorganic polyphosphate, amorphous nanoparticles

## Abstract

Initiated by studies on the mechanism of formation of the skeletons of the evolutionary oldest still extant multicellular animals, the sponges (phylum Porifera) have provided new insights into the mechanism of formation of the Ca-phosphate/hydroxyapatite skeleton of vertebrate bone. Studies on the formation of the biomineral skeleton of sponges revealed that both the formation of the inorganic siliceous skeletons (sponges of the class of Hexactinellida and Demospongiae) and of the calcareous skeletons (class of Calcarea) is mediated by enzymes (silicatein: polymerization of biosilica; and carbonic anhydrase: deposition of Ca-carbonate). Detailed studies of the initial mineralization steps in human bone-forming cells showed that this process is also controlled by enzymes, starting with the deposition of Ca-carbonate bio-seeds, mediated by carbonic anhydrases-II and -IX, followed by non-enzymatic transformation of the formed amorphous Ca-carbonate deposits into amorphous Ca-phosphate and finally hydroxyapatite crystals. The required phosphate is provided by enzymatic (alkaline phosphatase-mediated) degradation of an inorganic polymer, polyphosphate (polyP), which also acts as a donor for chemically useful energy in this process. These new discoveries allow the development of novel biomimetic strategies for treatment of bone diseases and defects.

## 1. Introduction: Evolution of the Metazoan Skeleton

With the elucidation of the functional genome of the sponges (phylum Porifera), using the demosponge *Suberites domuncula* as a model system [1], it became overt that the different metazoan taxa originate from one ancestor multicellular animal which is closely related to ancient sponge roots; this common ancestor evolved about 700 million years ago [2]. Even though several papers proposed that other taxa—like the Ctenophora—represent the basis of the Metazoa, a recent study confirmed our earlier conclusion [3]. Analyzing large genomic data sets revealed that “sponges (Porifera) are the sister-group to all other multicellular animals” [4] and proved “previous findings of trees with “Ctenophora-sister” were due to artifacts”. Furthermore, a recent statistical analysis revealed [5] that a few “innovative” genes can drive evolution [6]. An impressive example is the evolution of the integrin gene in sponges [7]; the expressed proteins allows the establishment of multicellularity.

The introduction of the molecular cloning of genes, which are—and this is imperative—coding for informative proteins, has rapidly increased our knowledge on the evolution of the multicellular animals from the common ancestor with the Porifera. In addition, it became possible to understand the steps of the evolution in this phylum, from the siliceous sponges with the class of Hexactinellida as the phylogenetically oldest one and the younger class of Demospongiae [8,9], to the calcareous sponges with the single class of Calcarea [10]. A recent study indicates that in addition of these three mentioned sponge taxa (Hexactinellida, Demospongiae, Calcarea) also a fourth can be pinpointed, the Homoscleromorpha; all four appear to have evolved at the same time [11].

It was surprising that these simple metazoan animals comprise almost all structural and functional metabolic circles, e.g., the kinases [12], the cell adhesion molecules [13], the Rhesus factor [14] or even the immunoglobulins [15], which are known in the “crown” metazoan taxa. In turn, we proposed a common metazoan “Bauplan” for all multicellular animals [1]. In earlier reviews a detailed summary of the functional aspects of the sponge circuits has been given (reviewed in Refs. [16,17,18]).

The major problems which had to be solved to understand the different facets of the complex Bauplan of the siliceous sponges and the calcareous sponges included the formation of their skeletons. Driven by general scientific approaches concerning the basic principles of metabolic processes in living systems, we asked for enzymes that are underlying and driving the inorganic mineralization in the two sponge taxa, the siliceous Demospongiae and calcareous Calcarea. A series of thorough studies disclosed the first enzyme, termed silicatein, involved in the deposition of the inorganic siliceous skeleton of *Tethya aurantia* [19] *S. domuncula* [20,21]. Silicatein catalyzes the polymerization of orthosilicate to polymeric biosilica. At the same time, it could be shown for the first time that enzymes are also functionally active in the formation of inorganic polymers from inorganic precursors. This work disclosed that sponge skeletal formation follows a genetically controlled hierarchical pathway, resulting in the formation of the picturesquely and intricately architectured spicules (reviewed in: [22]). Two isoforms of silicatein, silicatein-α and silicatein-β have been identified. The remarkable feature of these enzymes is the fact that they function not only as enzymes, forming covalent linkages, but are also structure-giving proteins that provide the platform for the organization of the silica spicules [23]. The biosilica formed during the enzymatically driven sol-gel process is a soft, gel-like inorganic polymer, catalyzed by a multi-protein system. In turn, this soft biosilica undergoes a biologically controlled process of syneresis, resulting in a shrinkage of the silica network [22]. Finally, biosilica is transformed into an elastic solid and gains the characteristic spicule morphology.

The next challenge that has been addressed was the elucidation of the formation of the calcareous skeleton. Again it was disclosed that it is an enzyme that forms those inorganic deposits. It is a carbonic anhydrase that is the initial driving molecule [24,25]. The cDNA encoding this enzyme has been cloned form the calcareous sponge *Sycon raphanus*. The skeletal elements of this sponge are composed of almost pure calcium carbonate. This mineral is formed in reactions that are catalyzed by this enzyme. In a biomimetic approach using a recombinant carbonic anhydrase, we succeeded to form amorphous pat-like particles that subsequently rearrange to crystalline rhomboid/rhombohedroid crystals with a dimension of about 50 µm. Surprising was the finding, based on light microscopical inspection and scanning electron microscopy studies, that the newly formed calcitic crystals associate with the calcareous spicules from *S. raphanus* in a highly ordered manner. Even more surprising was the observation that the calcitic crystals almost perfectly arrange in an array orientation along the two opposing planes of the spicules, leaving the other two plane arrays uncovered [25]. From those data it has been concluded that the carbonic anhydrase is the key enzyme that controls the calcium carbonate biomineralization process in Calcarea.

## 2. Composition of Vertebrate Bone: Ca-Carbonate and Ca-Phosphate Deposits

**Figure 1:**
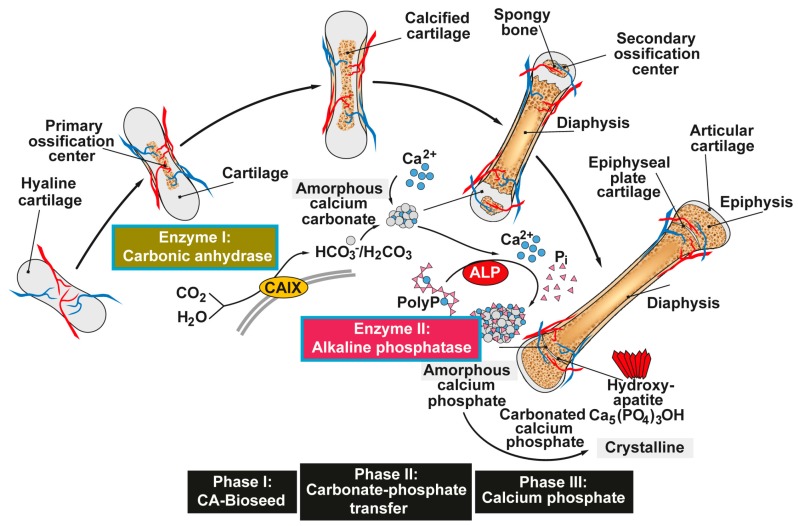
Proposed phases of bone mineral deposition. In endochondral ossification the hyaline cartilage begins to calcify, after the ingrowth of blood vessels, at the primary ossification centers in the diaphysis. Later, spongy bone is also formed in the epiphyses at the secondary ossification centers, with two regions of the hyaline cartilage remaining on the surface of the epiphysis (articular cartilage) and the epiphyseal plate (growth region) between the epiphysis and the diaphysis. The mineral deposition is driven by two enzymes (Enzyme I: carbonic anhydrase; and Enzyme II: alkaline phosphatase, ALP) and can be subdivided into three stages. *Phase I:* Carbonic anhydrase-mediated formation of amorphous Ca-carbonate bio-seeds. *Phase II:* Carbonate-phosphate transfer (non-enzymatic) using orthophosphate from ALP-mediated hydrolysis of polyphosphate (polyP). *Phase III:* Formation of amorphous (carbonated) calcium phosphate and finally crystalline hydroxyapatite. Modified according to [31]. (Reproduced with permission from the Royal Society of Chemistry, 2015).

Vertebrate bone, a biomineral, is composed of a mineral phase (Ca-deposits; approximately 60 to 70% *w/w*) and an organic matrix (mainly collagen; ≈20 to 30% *w/w*) and 10% of water (reviewed in [26,27]). It is well established that the process of mineralization in bone is a highly regulated process, driven by a tuned interplay between the bone-forming cells (osteoblasts) and the bone-resorbing cells (osteoclasts) organized by a complex organic extracellular (fibrillar) mesh of macromolecules forming a three dimensional porous scaffold; Figure 1. Furthermore, it has been well documented that during bone formation the extracellular matrix undergoes mineralization primarily around collagen fibrils that function as the basic building blocks of the bone. In addition to those fibrillar proteins, non-collagenous proteins act as second framework in a regulatory way during the mineralization process. To those non-collagenous proteins belongs the dentin matrix phosphoprotein 1, non-collagenous, acidic extracellular matrix protein that primarily regulates cellular morphogenesis events and differentiation processes [28]. Hydroxyapatite crystals become deposited on these phosphoprotein molecules if sufficient Ca^2+^ and phosphate units are supplied [29]. During this process Ca^2+^ deposit formation in vitro was found to be a sequential and stepwise process that starts with a rapid nucleation phase during which Ca^2+^ becomes bound to the phosphoprotein; the subsequent hydroxyapatite crystal formation takes days or weeks. Around the mineral deposition complex together with the osteoclasts, and odontoblasts, a dynamic intracellular Ca^2+^ balance is initiated and maintained that is controlled by various transmembraneous Ca^2+^ transport mechanisms, e.g., Ca-ATPase, Na^+^/Ca^2+^ exchangers and intracellular Ca^2+^-binding proteins (see Ref. [30]).

Very interesting to note is the fact that in vertebrates, besides of the hydroxyapatite-composed bones, biomineralized otoliths exist that are found in the vestibular labyrinth of the vertebrate ear. Surprisingly, they consist, besides of organic matrix proteins, to 90–95% of Ca-carbonate in the aragonite form [32,33]. As an organic matrix otolin, a collagenous protein, has been identified that comprises an important role during growth and function of otolith structures [34,35]. Furthermore, otoconins, Ca^2+^-binding proteins exist in the vestibular system, which initiate Ca^2+^-deposition [36].

Likewise, intricate and compelling is the fact that Ca-carbonate also co-exists with Ca-phosphate in the vertebrate bone [37]. Under physiological conditions, those Ca-carbonate deposits are formed primarily extracellularly, while pathological calcification of soft tissues predominantly occurs intracellularly [38]. Bone formation is based on a tightly controlled process between osteoblasts and fibrillar organic structures, starting from collagen fibrils around which poorly crystalline carbonated apatite crystals are deposited as carbonate-apatite [39,40]. Supportive spectroscopic studies suggested that Ca^2+^-deposition in osteoblasts starts intracellularly in calcium-containing vesicles which substantially contribute to the bone apatite formation [40]. Finally, amorphous Ca-phosphate particles are formed and released from the cells in close association with collagen fibrils that form the matrix for the subsequent crystallization process. Alternatively, it has been suggested that the extracellular fluid is sufficiently saturated with respect to Ca^2+^ and phosphate, perhaps as Ca^2+^-polyphosphate (Ca-polyP) [41] promoting Ca-phosphate deposition [42].

## 3. Ca-Carbonate Bio-Seeds Initiate Ca-Phosphate Deposits: Carbonic Anhydrase

Both Ca-phosphate formation [42] and Ca-carbonate deposition [43] are exergonic processes. Therefore, it has been pressing for us to ask the question if enzymatic processes are involved in the initiation and control of those mineralization processes. Based on our recent studies [24,25] we initiated studies to elucidate if the initial mineralization in bone-mineralizing cells is also controlled by enzymes [44]. In the first series of experiments we presented experimental evidence that Ca-phosphate/hydroxyapatite crystals formation starts, in human SaOS-2 cells, with the deposition of amorphous Ca-carbonate deposits (Figure 1). This reaction is enzymatically controlled primarily by the carbonic anhydrase-II. The product amorphous Ca-carbonate acts as a positive actor during in vitro mineralization. In a subsequent series of experiments, it has been studied which carbonic anhydrase becomes upregulated under physiological normoxic condition (8% versus ambient (21%) oxygen tensions). Those gene expression studies favorized the carbonic anhydrase-IX as the dominant enzyme that forms the Ca-carbonate bio-seeds [45]. From those Ca-carbonate bio-seeds the mineralization process progresses further to Ca-phosphate.

## 4. Transformation of Ca-Carbonate to Ca-Phosphate Deposits: Non-Enzymatic Step

In a comprehensive review solid experimental evidence has been presented documenting that the amorphous phases are the precursors for any kind of crystalline deposits in animals [46]. This also includes bone mineral, Ca-phosphate, formation. In view of, at that time, existing data we successfully performed in vitro experiments to prove the concept that amorphous Ca-carbonate is transformed non-enzymatically into amorphous Ca-phosphate [47]. For the experiments amorphous Ca-carbonate and the different crystalline phase of Ca-carbonate (vaterite, aragonite and calcite) were exposed to inorganic phosphate at different concentrations. Applying FTIR spectroscopy it could be shown that amorphous Ca-carbonate is processed to vaterite, aragonite and calcite in dependence on the concentration of phosphate. Furthermore, these data suggested that also in vivo these reactions are passed through [47].

## 5. Origin of the Phosphate in Bone Mineral: Inorganic Polyphosphate

**Figure 2:**
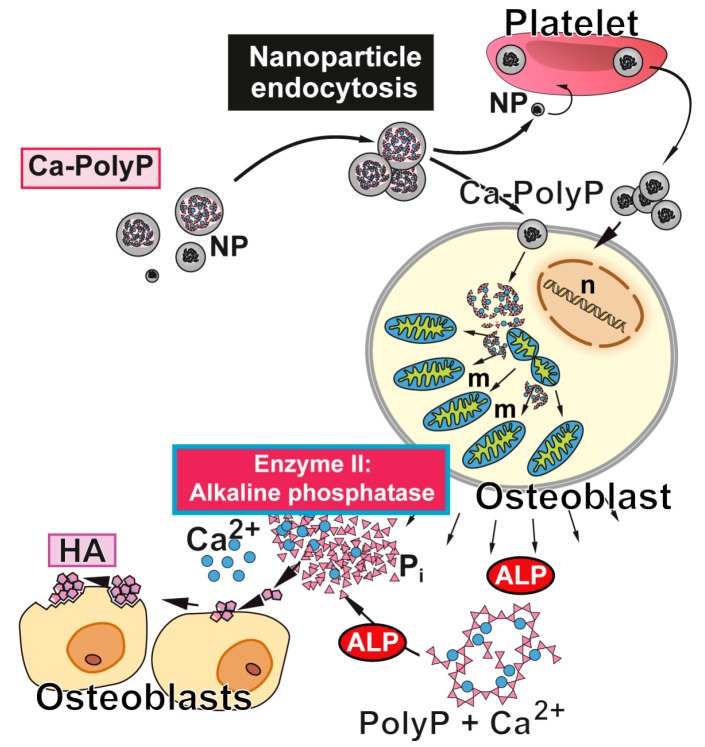
Biological function of polyP and Ca-polyP nanoparticles as donor of metabolically useful energy during bone mineral formation. The nanoparticles (NP) are taken up (and released) by platelets, which are able to accumulate high amounts of polyP, and/or taken up by bone cells. In osteoblasts, polyP induces an increase in the number mitochondria (m) and the intracellular level of ATP [53]. After being released into the extracellular space, polyP is hydrolyzed by ALP (Enzyme II). The released orthophosphate and Ca^2+^ are used for hydroxyapatite (HA) synthesis. n, cell nucleus. Modified according to [54]. (Reproduced with permission from the John Wiley, 2015).

Interestingly enough we found that sponges with the examples of the siliceous sponges Ephydatia muelleri and Tethya lyncurium contain inorganic polyphosphate (polyP) that is involved in the control of growth control and mineralization and contain enzymes that are able to degrade this polymer [48,49,50]. Then, besides of carbonate, phosphate is a major buffer system under physiological conditions. In addition, phosphate exists in a polymerized state, as inorganic polyP both in a free state in serum and intracellularly in blood platelets (reviewed in Ref. [51]); Figure 2. The interesting feature of polyP is that besides (potentially) providing phosphate units for Ca-phosphate mineralization, this polymer delivers chemically useful energy during enzymatic hydrolysis using the enzyme alkaline phosphatase (ALP; [52]). In turn it has been experimentally demonstrated that cells exposed to polyP react with an increased intra- and also extracellular ATP and ADP pool [53]. Based on these data, a new concept has been developed that highlight polyP not only as a phosphate source but also as a donor for metabolically available energy, especially in the extracellular space (for a review, see Refs. [54,55]).

**Figure 3:**
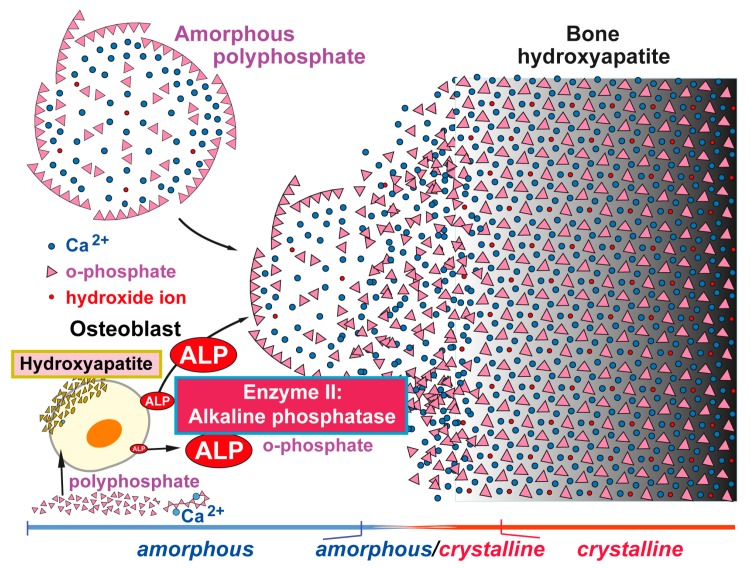
Formation of amorphous Ca-phosphate and finally crystalline hydroxyapatite induced by amorphous biologically active (morphogenetically active) nano/microparticles consisting of polyP salts, e.g., Ca-polyP. This polymer enhances the expression and activity of the ALP (Enzyme II) in osteoblasts [58]. Degradation of polyP by the ALP provides orthophosphate and calcium ions, which serve a substrate for calcium phosphate/hydroxyapatite formation. The initially formed amorphous calcium phosphate is then converted into the crystalline form. Modified according to [60]. (Reproduced with permission from Elsevier, 2016).

**Figure 4:**
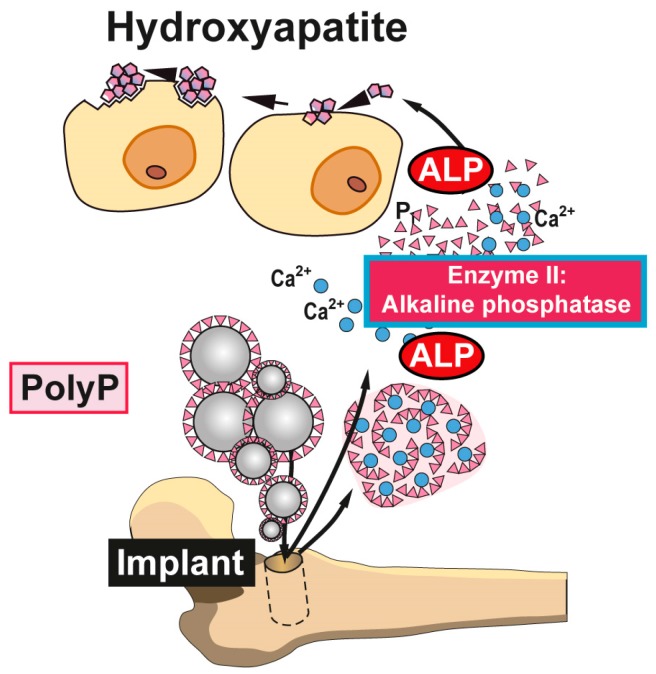
Biomedical application of polyP. Nanoparticles or microparticles prepared from polyP salts can be used as a scaffold material for bone implants. It is expected that after implantation into the bone defect, these nano/micoparticles attract and induce differentiation/proliferation of osteoblast precursors to mature functionally active (hydroxyapatite-forming) osteoblasts. Disintegration of the morphogenetically active amorphous particles and degradation of Ca-polyP by osteoblast-associated ALP to orthophosphate (and calcium) ions support new bone formation in the implant region. Animal studies confirmed the enhanced regeneration of bone tissue with Ca-polyP implants; modified after [54]. (Reproduced with permission from the John Wiley, 2015).

In a biomimetic approach we succeeded to fabricate polyP into amorphous nanoparticles, similar to those found in vivo; e.g., Refs [56,57]; Figure 3. With those encapsulated particles it became possible to accelerate bone mineral formation in vitro [58] as well as in vivo [59]; Figure 4.

## 6. Reaction Chain from Ca-Carbonate Bio-Seeds to Ca-Phosphate-Based Hydroxyapatite

These findings allowed us for the first time to outline the pathway of bone mineral formation including enzymes that control crucial steps during Ca-phosphate/hydroxyapatite formation, starting at the stage of amorphous Ca-carbonate bio-seeds [61]; see Figure 3. The gathered findings show that the initial Ca-carbonate bio-seeds are synthesized by carbonic anhydrases-II and -IX. This mineral then undergoes transformation, in the amorphous state, to Ca-phosphate, a mineral that maturates to hydroxyapatite. These findings provide a rational and sound platform for the development of biocompatible osteogenic scaffolds for bone repair [59]. In addition, both enzymes involved in human bone formation, the carbonic anhydrase and the ALP, provide novel potential targets for drugs that might be beneficial for therapy of human bone diseases like osteoporosis.

## 7. Contribution of the Knowledge on Sponge Skeleton for Biomedicine

The studies with siliceous and calcareous studies disclosed that—as a rule—also inorganic depositions in Metazoa are the result of enzymatic processes. There are the silicatein(s) in the siliceous sponges that are the drivers of the inorganic silicification of the skeleton (reviewed in: [62,63]) or the carbonic anhydrase(s) that are synthesizing the calcareous skeletons in the Calcarea [25,64]. Asking the question if also in the “crown taxon” the human calcium carbonate is formed during the hydroxyapatite-based bone formation we disclosed that also there the carbonic anhydrase(s) are involved in biomineralization and more precisely during the initial phase of bio-seed formation [65]. A striking underlining of this finding was the discovery that a sponge metabolite, quinolinic acid, was able the activate carbonic anhydrase and in turn also increases the mineralization potency of human bone cells [66].

Accepting that evolution drives in a conserved manner the progress of animals from the more simple to the more complex state allowed the formulation of new strategies even to decipher the complex mineralization stages of bone formation. From studying the skeleton of the siliceous sponges the knowledge was obtained that—basically as expected, but not clarified—even inorganic minerals are formed enzymatically. Progressing further to the calcareous sponges it became overt that it is Ca-carbonate and not Ca-phosphate from which mineralization of hydroxyapatite starts. Even more, the data obtained introduced the new concept that the abundantly present inorganic polymer polyP serves, after enzymatic hydrolysis via the ALP, as donor for the phosphate components required for bone formation and also as energy supply for extracellular endergonic reactions. The discovery of polyP as an extracellular system for the storage and delivery of metabolically useful energy may also contribute to the understanding of other energy-dependent processes occurring in the extracellular space and may progress the development of further applications based on this inorganic polymer in the biomedical field, in addition to bone repair and regenerative therapy of the bone diseases.
